# Core Temperature in Triathletes during Swimming with Wetsuit in 10 °C Cold Water

**DOI:** 10.3390/sports7060130

**Published:** 2019-05-28

**Authors:** Jørgen Melau, Maria Mathiassen, Trine Stensrud, Mike Tipton, Jonny Hisdal

**Affiliations:** 1 Institute of Clinical Medicine, University of Oslo, 0316 Oslo, Norway; jonny.hisdal@medisin.uio.no; 2 Department of Vascular surgery, Oslo University Hospital, 0424 Oslo, Norway; 3 Prehospital Division, Vestfold Hospital Trust, 3103 Toensberg, Norway; 4 Department of Cardiology, Telemark Hospital Trust, 3710 Skien, Norway; maria.mathiassen@gmail.com; 5 Department of Sports Medicine, Norwegian School of Sport Sciences, 0806 Oslo, Norway; trine.stensrud@nih.no; 6 Extreme Environments Laboratory, Department of Sport and Exercise Science, University of Portsmouth, Portsmouth PO1 2ER, UK; michael.tipton@port.ac.uk

**Keywords:** swimming, core temperature, skin temperature, wetsuit, triathlon, endurance

## Abstract

Low water temperature (<15 °C) has been faced by many organizers of triathlons and swim-runs in the northern part of Europe during recent years. More knowledge about how cold water affects athletes swimming in wetsuits in cold water is warranted. The aim of the present study was therefore to investigate the physiological response when swimming a full Ironman distance (3800 m) in a wetsuit in 10 °C water. Twenty triathletes, 37.6 ± 9 years (12 males and 8 females) were recruited to perform open water swimming in 10 °C seawater; while rectal temperature (Tre) and skin temperature (Tskin) were recorded. The results showed that for all participants, Tre was maintained for the first 10–15 min of the swim; and no participants dropped more than 2 °C in Tre during the first 30 min of swimming in 10 °C water. However; according to extrapolations of the results, during a swim time above 135 min; 47% (8/17) of the participants in the present study would fall more than 2 °C in Tre during the swim. The results show that the temperature response to swimming in a wetsuit in 10 °C water is highly individual. However, no participant in the present study dropped more than 2 °C in Tre during the first 30 min of the swim in 10 °C water.

## 1. Introduction

Long distance triathlon is rising in popularity [1]. In 2003, the first “Norseman Xtreme Triathlon” was arranged in Norway, and the race soon became known as one of the toughest triathlons in the world [2]. Athletes swim 3800 m in the Hardangerfjord, bike 180 km with approximately 3000 m of vertical ascent and then run 42 km, to finish at the peak of Mt. Gaustadoppen at 1883 m above sea level [3]. The low water temperature (<15 °C) has generally been a challenge for the organizers. In 2015, the participants faced a water temperature of 10 °C, and the swim was then shortened to half the distance [4]. 

Low water temperature has been faced by many organizers of triathlons [5] and swim-runs [6] in the northern part of Europe during recent years, and more knowledge about how cold water affects athletes swimming in wetsuits is warranted.

The International Triathlon Union (ITU) has taken this into account in their regulations of racing water temperature and wetsuit usage in ITU sanctioned races [7]. Recently, scientific inquiries into the rationale behind these regulations have been made, and the rules have been modified accordingly [8]. The International Swimming Federation (FINA) has specified 16 °C as their lowest water temperature in their Open Water Swimming Rules [9]. In a recent study [8], Saycell J, Lomax M, Massey H, et al. identified lean swimmers and cold water as significant risk factors for hypothermia. This has also been elucidated further, with new minimum water temperature limits for open water marathon swim racing [10]. 

Despite this, the knowledge of how deep body temperature is affected in triathletes swimming in wetsuits in cold water down to 10 °C is limited. For the vast majority of triathletes, the swim portion is completed in <2 h.

The aim of this study was therefore to investigate the physiological response to swimming in a wetsuit in 10 °C water. Based on previous experience, our hypothesis was that the deep body temperature (Tre) would decrease less than 1 °C·h^−1^ during swimming in 10 °C water with a properly fitting wetsuit, suggesting that the Tre would not drop more than 2 °C (or below 35 °C) during a full swim in an Ironman competition.

## 2. Materials and Methods

### 2.1. Participants

The study protocol was evaluated by the Regional Ethics Committee (REC) (ref 2015/1533/REK Sør-Øst), according to the principles of the declaration of Helsinki. Before inclusion, all participants provided written informed consent. Twenty participants (12 males, 8 females) were recruited for the present study. All were active triathletes, at elite- or recreational level. Recruitment took place via social media, and the individuals had to be able to swim 3800 m non-stop in less than 1h and 45 min, not have any history of cardiovascular disease or arrhythmias and have their own wetsuit.

### 2.2. Measurements

Prior to the tests, medical screening was performed by the study doctor and a nurse. The screening included a medical survey and an ECG test (Cardiovit AT102 Plus, Schiller Handelsgesellschaft m.b.H., Sveits) in accordance with the recommendation of the European Society of Cardiology [11,12].

Baseline measurements, including weight, height, DXA-scan (Lunar Prodigy densitometer, GE Medical Systems, WI, USA) were performed 2 h before the start of the swim at the Norwegian School of Sport Sciences (NIH) in Oslo. Maximal oxygen uptake (VO_2max_) was measured at NIH, within one week after the test by a Oxycon Pro analyzer (Jaeger Instrument, Carefusion/BD, San Diego, CA, USA) using a graded (5.3%) running test on a treadmill (Bari-Mill, Woodway, WI, USA) with gradually increasing running speed each minute until exhaustion, according to Astrand, Rodahl et al. 2003 [13].

All participants had a warm-up of easy running (10 min) on the treadmill before the test started. During the test, all participants wore a nose clip (9015 Reusable Series, Hans Rudolph Inc., Kansas City, MI, USA) and used a silicone rubber mouthpiece (9060 Reusable Series, Hans Rudolph Inc., Shawnee Mission, KS, USA).

VO_2max_ was identified when a plateau (a rise of less than 2 mL·kg^−1^·min^−1^ in VO_2_, despite increasing running speed) was observed. In addition, two more criteria of VO_2max_ were applied, a respiratory exchange ratio (*RER*) > 1.05 and heart rate of >95% of maximum heart rate.

After testing, the participants were transferred to the test site in the Oslofjord at Høvik, 20 min outside of Oslo city, where the temperature sensors were mounted on the participants. A skin sensor (YSI 400) was mounted on the upper left side of the chest (approximately 8 cm below *clavicula*) and a rectal probe (YSI 400, YSI Incorporated, Yellow Springs, OH, USA) was self-inserted by the athletes after instruction from the scientists. The rectal probe was inserted 10 cm past the anal sphincter. The sensors were connected to a logging device (Veriteq Spectrum Precision Thermistor Logger 1400, Surrey, BC, Canada) and temperatures were logged every minute from 15 min prior to the swim until a minimum of 45 min after the swim. No rewarming intervention was incorporated in this study. The logger was mounted in a custom-made waterproof box (length 12 cm, width 7 cm and height 4 cm) that was taped to the back of the outside of the participant’s wet suit. The logging system did not affect swimming technique.

### 2.3. Swim Test

The testing was very time consuming, and due to safety reasons, we were not able to have more than one test subject in the water at a time. The swim test was therefore performed over a period of three consecutive days. Mean (SD) water temperature was 10.0 (0.7 °C) and air temperature 7.4 (2.1 °C) during the three test days. On day one, six participants swam 3800 m (82 (14) min), and on day 2 and 3, the swim time was shortened to a maximum of 55 min. In total, 13 participants performed 46 (5) min of swimming. To ensure the optimal fit of the wetsuit, the participants used their personal wetsuits, approved in accordance with the ITU Competition Rules for triathlon [7]. The thickness of the wetsuit should not exceed 5 mm of thickness anywhere, and have long arms and legs. In addition, a standard silicone swim cap was used, with no other aid for warming the body during the swim. During the first day, six participants were tested, and all of them swam a full Ironman distance (3800 m). After the first day of testing, we observed a rectal temperatrue (Tre) below 35 °C in one of the participants, and we therefore decided to reduce the swim time to a maximum of 55 min the next two days to prevent a fall in Tre below 35 °C. In none of the athletes who participated in the last 2 days of testing did the Tre fall below 35 °C. The participants were swimming one at a time, a maximum of five meters from the pier and were constantly monitored by five paramedics and a rescue swimmer. A medical doctor was present at the test site at all times during the three days of testing. All rescue personnel where updated and trained in the latest protocols regarding hypothermia [14] and advanced cardiopulmonary resuscitation [15]. Mandatory rescue- and medical equipment was located on the pier for the paramedics and medical doctor to use if needed [16].

### 2.4. Data Analysis and Statistics

The study was powered to be able to detect a drop in core temperature >0.5 °C during the swim. Given a significance level of 0.05 and a power of 80%, 16 participants were needed, given a start temperature at 37.5 ± 0.5 °C. Further, to compensate for a 20% dropout rate, a total of 20 participants were recruited to the study. Statistical analyses and all graphics were performed in SigmaPlot 10.0 (Systat Software, Inc., GmbH, Erkrath, Germany). Pearson Product Moment Correlation was performed to evaluate correlation between variables. Data are reported as mean (standard deviation) unless otherwise stated. A *p*-value <0.05 was considered statistically significant.

## 3. Results

One participant was excluded before swimming due to failing the medical screening, and in two participants, Tre was not recorded during the swim due to equipment failure. Seventeen participants (6 women) were therefore included in the final analysis (Table 1).

### 3.1. Rectal Temperature (Tre)

**Figure 1:**
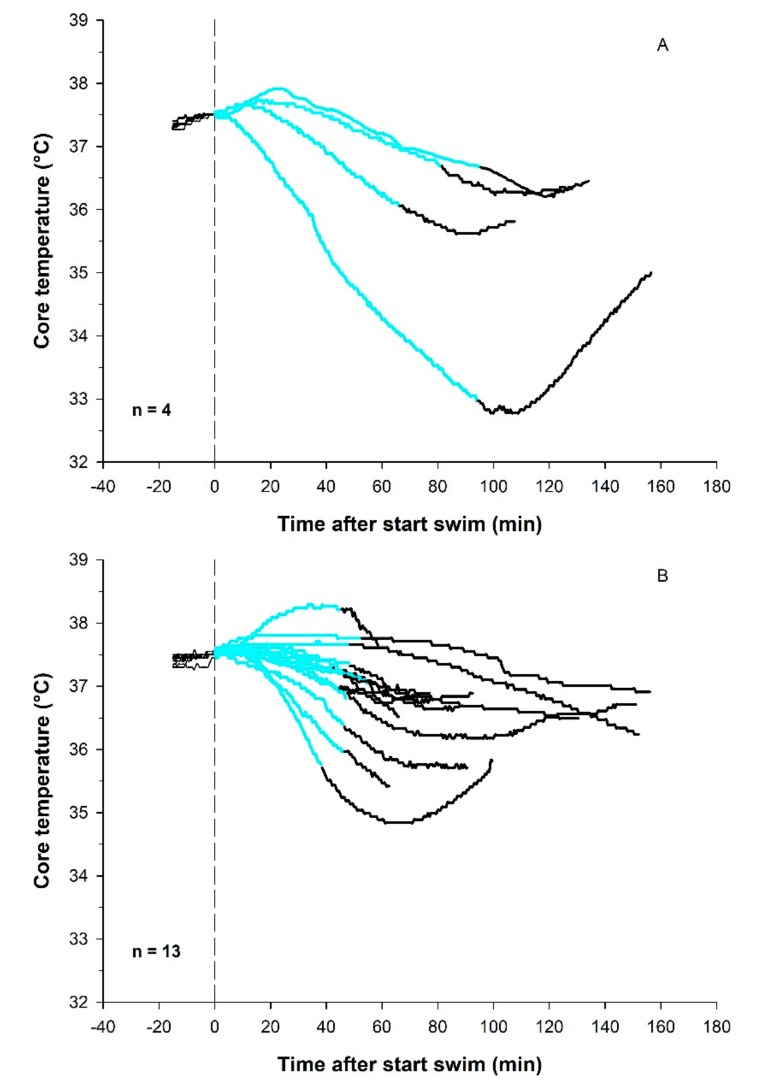
Tre before (black line), during (blue line) and after (black line) swimming in 10 °C water. Panel A shows results for the athletes that swam 3800 m in 82 (14) min (n = 4), and panel B shows results after the shortened swim to 46 (5) min (n = 13). For comparison, all temperature curves are adjusted to start at 37.5 °C at swim start.

Before the swim, average Tre was 36.6 (0.1) °C. The Tre of all participants was maintained for the first 10 min of the swim. In 13 of the 17 participants, Tre dropped below starting value during the swim, with a statistically significant drop in Tre of 0.9 (1.1) °C in the group (*p* < 0.001). For all 13 participants that displayed a fall in Tre, a further fall (“afterdrop”) in Tre was observed after the swim (0.6 (0.3) °C). The average (SD) time from exiting the water until lowest temperature was 25 (12) min. Tre for the participants that swam 3800 m (n = 4) are displayed in Figure 1, panel A, and panel B shows results for 13 athletes that swam for a maximum of 55 min.

**Figure 2:**
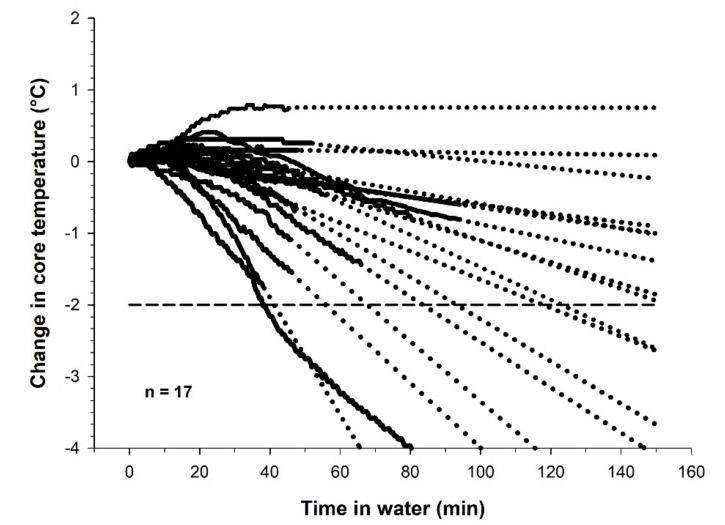
Solid line shows the development of Tre during swimming in 10 °C cold water. Dotted lines show extrapolated time course, based on the slope for Tre during the last 20 min of the swim (n = 17).

The slope for the drop in Tre was on average 1.38 (1.24) °C·h^−1^. The results show that with an exposure time of 135 min, 47% (8/17) of the athletes would experience a drop in Tre larger than 2 °C (Figure 2). However, at 30 min of swim time, none of the participants in the present study experienced a drop in Tre >2 °C.

### 3.2. Skin Temperature (Tsk)

**Figure 3:**
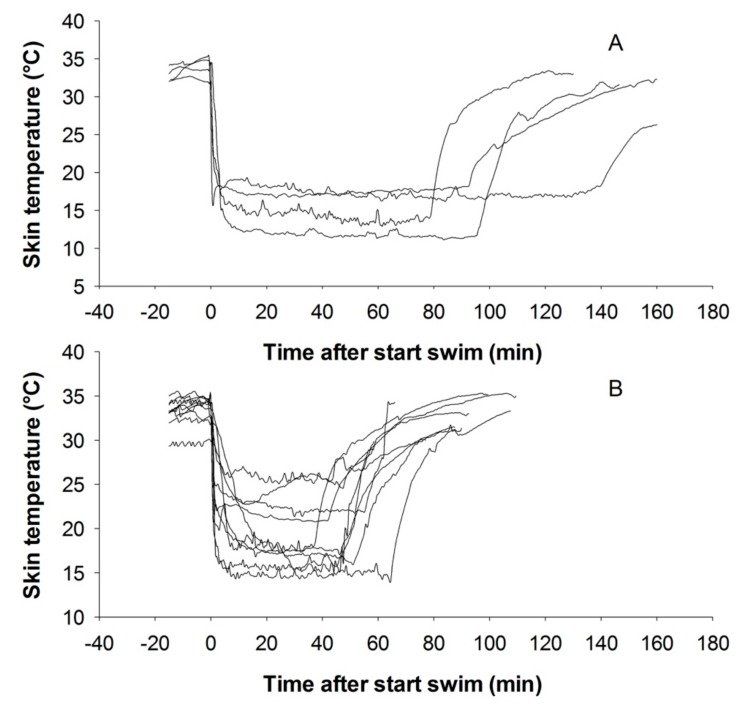
Tsk before, during and after swimming in 10 °C water with a wet suit. Panel A shows results for the 4 athletes that swam 3800 m in 82 (14) min (n = 4), and panel B shows results for 10 athletes that swam a shortened swim to 46 (5) min (n = 10).

Due to technical problems with the skin sensors on three of the athletes, Tsk was successfully recorded during the swim in 14 of 17 athletes where Tre were recorded. Average Tsk beneath the wet suit was 33.3 (0.3) °C before the swim and was significantly reduced to 19.2 (1.7) °C during the first 30 min of the swim (*p* < 0.001). Tsk before, during and after the swim for all athletes are shown in Figure 3.

### 3.3. Relation between Tre, Skin Temp, fat% and Gender

**Figure 4:**
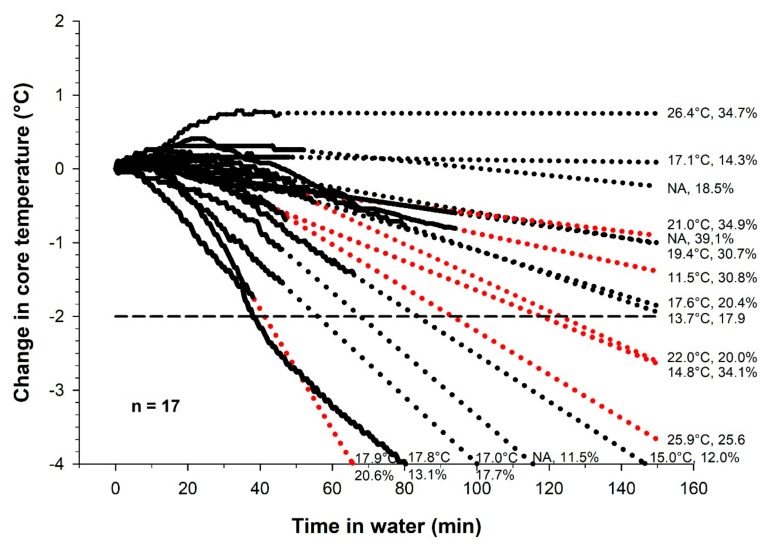
Solid line shows the Tre during swimming in 10 °C water. Dotted lines show the linearly extrapolated time course, based on the slope for Tre during the last 20 min of the swim (red dots = female). Average Tsk during swim and body fat % are presented for all participants (n = 17).

We observed a significant correlation between the slope for Tre during the swim and total fat mass (kg), (r^2^ = 0.25, *p* = 0.04). There was a non-significant tendency for correlation between the slope for Tre during the swim and % bodyfat (%), (r^2^ = 0.21, *p* = 0.06) and BMI (r^2^ = 0.13, *p* = 0.08). No other significant correlations were observed between the slope for Tre during the swim and any of the other following relevant variables as; weight (*p* = 0.33), height (*p* = 0.33), age (*p* = 0.51), LBM (*p* = 0.94), average skin temp last 20 min of swim (*p* = 0.86), hours swimming training per week (*p* = 0.47) or gender (*p* = 0.43). In Figure 4, change in Tre, Tsk and, fat% and gender are shown for all participants.

## 4. Discussion

The main finding in the present study was the heterogeneity in the temperature response to swimming in a wetsuit in cold water. However, for all participants, the Tre was maintained for the first 10–15 min of the swim, and no participants dropped more than 2 °C in Tre during the first 30 min of swimming in 10 °C water. However, given a swim time above 135 min, 47% (8/17) of the participants in the present study would be predicted to have greater than a 2 °C in Tre.

### 4.1. Rectal Temperature

The results from the present study showed that the participants were able to maintain the Tre for the first 10–15 min of the swim. An explanation for this is the cold-induced vasoconstriction at the skin’s surface, and the time required to set up a conductive cooling gradient from the water to the deep body tissues. The conductive cooling gradient is dependent on the length of the conductive pathway (size/fatness of the individual) [17]. Further, after this initial period, Tre started to drop in 76% (13/17) of our test participants. The linear pattern of the temperature curve, made it possible to calculate a slope, and therefore the possibility to interpolate the curves and predict Tre if swimming had been prolonged. Several studies have shown the potential harmful effects of hypothermia [18,19,20]. One of the study participants in the present study had a Tre as low as 33.1 °C, classified as mild hypothermia. When this was discovered, we immediately took action to prevent similar cases, and the exposure time to cold water was therefore reduced during days 2 and 3 of the project.

The results from the present study displayed a large heterogeneity in the Tre response. One participant started to drop in Tre after 10.5 min, and another increased in Tre during the swim. The participant with the early drop had a body fat % of 13.1, and the one that increased had a fat % of 34.7. Further analysis also confirmed a significant correlation between low body fat % and drop in Tre. This is in line with previous findings in other studies [8,17]. This should be of interest for race organizers, as more elite athletes often have a lower body fat % and therefore are more prone to become hypothermic during swimming.

It is complicated to prescribe safe limits for swimming in cold water due to the interaction between many variables that may affect the cooling rate [8]. In addition to the absolute water temperature: exposure time, metabolic heat production, body composition, body mass and wetsuit construction (length and thickness) and fit may affect the cooling rate. One important research question in the present study was to estimate how long it would take before the athletes reached a Tre of 35 °C or below. Figure 2 shows an estimation of this, where we have extrapolated the Tre cooling curves to predict when Tre exceeds a 2 °C fall. The cut-off for the swim in Norseman Xtreme Triathlon is 135 min. The average swim time during the last 10 years was approximately 82 min. The fastest athletes completed the swim in 50 min. The results from the present study show that given a water temperature at 10 °C, 47% of the athletes that swam for 135 min would drop more than 2 °C in Tre.

Given a well-fitted wetsuit, our results indicate that to avoid hypothermia, the exposure time should be limited to a maximum of 30 min, in 10 °C water. For the slowest swimmers, this would probably correspond to a maximum swim distance of 1000 m under such conditions.

### 4.2. Tsk

The results from the present study showed that the Tsk dropped immediately on entering the cold water and stabilized at a constant level within a few minutes. A relatively large variation in Tsk was observed between the participants during the swim (12–26 °C), however no significant relationship between the drop in Tsk and Tre was observed.

In the present study, the athletes used their own personal wetsuit of different brands, thickness and fit and this could possibly be the explanation for the lack of correlation between Tsk and drop in Tre. Evidence suggests a relationship between wetsuit fit and cardiovascular response [21]. The relationship between drop in Tre, Tsk and type and fit of wetsuit needs to be elucidated in further studies.

### 4.3. Post-Immersion Cooling

The post-immersion cooling observed in our study was on average 0.6 °C, and the lowest temperature was observed on average 25 min after the swim. The fact that Tre may continue to fall post- open water swim should be of interest to organizers. It is also important for triathlon organizers and triathletes to expect that Tre can fall in T1 (Transition Zone 1—the shift from swimming to cycling during a triathlon) and during the first part of the cycling [8]. Race organizers and medical crew should have increased levels of alertness during these periods. Our findings on post-immersion cooling is also in accordance with previous published results from Nuckton et al. 2000 [22] who studied open water swimmers in 11.7 °C water. In that study, post-immersion cooling was observed in 10 of 11 test participants. The effect is possibly worsened by the fact that triathletes are affected by the wind chill factor [23,24] during cycling (continued cooling). The International Triathlon Union (ITU) has taken this into consideration, as they have incorporated both air temperature and water temperature into their competition rules [7]. According to ITU competition rules, the swim can be shortened or cancelled according to a combined water temperature and air temperature.

### 4.4. Practical Implications

From a safety perspective, athletes competing in a race should never be exposed to environmental conditions that induce mild hypothermia or worse. The Tre therefore should not drop more than 2.0 °C, or below 35 °C. Taking into account the post-immersion cooling, the maximum drop during the swim should be less than 1.5 °C to ensure athletes’ body temperatures do not fall within hypothermic ranges during subsequent portions of the event. For those undertaking an open water swim only, it should be realised that the participants may have their lowest deep body temperature after the event when attempting, for example, to drive home.

### 4.5. Limitations

For practical reasons, the Tre continued to be measured 20–90 min after the swim. Ideally, the measurements should have been continued until the Tre was back to baseline values. Further, more details about the wetsuit (thickness, fit, conditions) is warranted. The surface temperature of the wet suit should also be measured to better explain the relationship between Tsk and the drop in Tre.

## 5. Conclusions

It is concluded that the temperature response to swimming in a wetsuit in 10 °C water is highly individual. However, the Tre of no participant in the present study cooled more than 2 °C during the first 30 min of the swim. To be on the safe side, this would probably correspond to a maximum swim distance of 1000 m in 10 °C water. One would expect even the least able swimmers to cover 1000 m in 30 min: with the caveat that they do not suffer swim failure due to neuromuscular cooling.

## Figures and Tables

**Table 1:** Demographic, anthropometric and physiological characteristics of the study sample; as a total and for both women and men separately. Values are given as mean ± SD.

	Total	Women	Men
**Number (n)**	17	6	11
**Age (yrs.)**	37.6 ± 9.0	37.5 ± 10.3	37.6 ± 8.8
**Body composition** **Weight (kg)** **Height (cm)** **LBM (kg)** **%BF (%)** **FM (kg)**	77.9 ± 7.4 177.6 ± 7.4 58.3 ± 11.5 23.3 ± 9.0 17.2 ± 7.4	66.4 ± 8.0 173.4 ± 5.6 46.2 ± 5.4 27.7 ± 6.6 17.8 ± 5.3	84.3 ± 12.9 179.9 ± 7.3 65.0 ± 7.8 20.9 ± 9.6 16.7 ± 8.6
**VO_2max_** **Relative (mL** **·** **kg^−1^** **·** **min^−1^)** **Absolute (L** **·** **min^−1^)**	57.5 ± 11.0 4.5 ± 1.1	49.3 ± 6.6 3.3 ± 0.5	62.4 ± 10.3 5.2 ± 0.7
**Training per week (hh:min)** **Total** **Swimming pool**	9:30 ± 4:06 1:36 ± 1:18	8:18 ± 4:54 1:36 ± 1:24	10:18 ± 3:42 1:36 ± 1:12

LBM is lean body mass; %BF is percentage body fat; FM is fat mass and VO_2max_ is maximal oxygen uptake.
